# Spatial segmentation and feature selection for desi imaging mass spectrometry data with spatially-aware sparse clustering

**DOI:** 10.1186/1471-2105-13-S18-A8

**Published:** 2012-12-14

**Authors:** Kyle D Bemis, Livia Eberlin, Christina Ferreira, R Graham Cooks, Olga Vitek

**Affiliations:** 1 Department of Statistics, Purdue University, West Lafayette, IN, USA; 2 Department of Chemistry, Purdue University, West Lafayette, IN, USA; 3 Department of Computer Science, Purdue University, West Lafayette, IN, USA

## Background

Recent experimental advances in matrix-assisted laser desorption/ionization (MALDI) and desorption electrospray ionization (DESI) have demonstrated the usefulness of these technologies in the molecular imaging of biological samples. However, development of computational methods for the statistical interpretation and analysis of the chemical differences present in the distinct regions of these samples is still a major challenge. In this poster, we propose statistically-minded methods and computational tools for analyzing DESI imaging experiments. Specifically, we present techniques for signal processing and unsupervised multivariate image segmentation, which are also applicable to other imaging mass spectrometry (IMS) methods such as MALDI.

## Method

Signal processing of DESI spectra typically involves binning to reduce dimensionality, but this inefficient for downstream analysis as it retains empty regions of the mass spectrum. In our proposed processing step, we apply a novel peak picking algorithm based on windowed smoothing splines that allows adaptive resolution based on spectral profile. With this approach, peaks are aligned using a recursive dynamic programming algorithm which accounts for the heterogenous nature of IMS data by making pairwise alignments between pixels based on their proximity. Peaks are then normalized using total ion count.

In order to segment the sample into sub-regions of homogenous chemical composition in MALDI images, Alexandrov & Kobarg [1] proposed two efficient spatially-aware clustering techniques. We demonstrate that these approaches are also useful for DESI. Moreover, we extend one of these clustering methods using statistical regularization techniques that enable simultaneous feature selection of structurally-important peaks and facilitate interpretation.

## Conclusions

**Figure 1:**
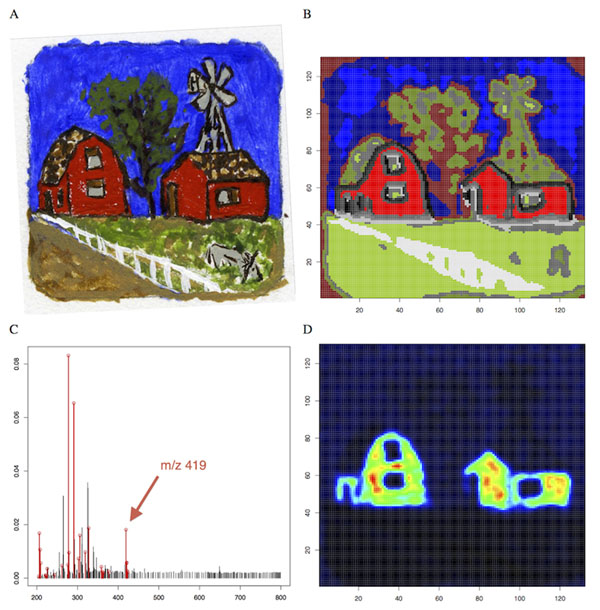
(A) Optical scan of a painting as an example of a non-biological sample and (B) the results of spatially-aware sparse clustering with C = 15 clusters. The discovered sub-regions often match up with the known colors used in the painting, but also shows regions that could not be accurately distinguished, such as the gray donkey in the lower right. (C) The mean mass spectrum of the cluster corresponding to the red houses with the 27 structurally-important ions selected via regularization for s = 0.2 marked in red and (D) the ion image for one such peak at m/z 419.

**Figure 2:**
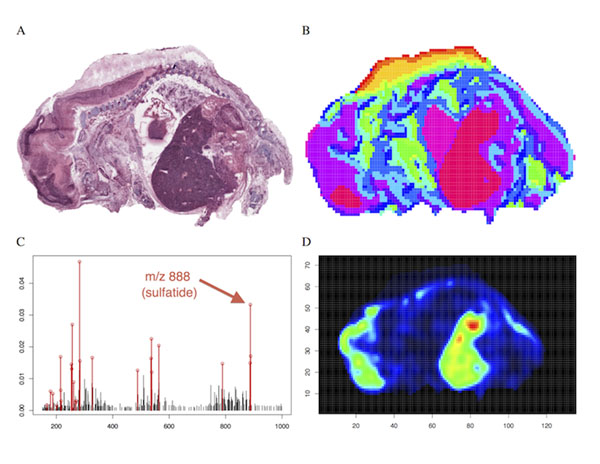
(A) The fetal pig section and (B) the results of spatially-aware sparse clustering with C = 15 clusters. Note that the clusters are regions of homogenous chemical composition and often match up with visually distinguishable anatomical features, while also revealing possible hidden chemical structures in regions that are visually ambiguous. (C) The mean mass spectrum of the cluster corresponding to the liver and part of the brain with the 25 structurally-important ions selected via regularization for s = 0.6 marked in red and (D) the ion image for one such peak at m/z 888.

We evaluate the performance of the proposed methods in two applications. First, in a non-biological application of DESI-imaging, we recreate a painting from the clustering of its DESI mass spectra (Figure 1). Since the visual content of the painting is known, it can be used as a gold standard to evaluate the performance of these methods. In the second application, we present the spatial segmentation of a fetal pig section, and evaluate the performance of our methods by the quality of the mapping between the spatial segmentation and the morphological and functional structures (Figure 2). We show that statistical regularization improves accuracy and interpretation of the spatial segmentation over existing approaches.

## References

[B1] Alexandrov T Kobarg JH Efficient spatial segmentation of large imaging mass spectrometry datasets with spatially aware clustering Bioinformatics 2011 27 13 i230 238 10.1093/bioinformatics/btr246 21685075 PMC3117346

